# Challenges of establishing an emergency medical team in Papua New Guinea

**DOI:** 10.5365/wpsar.2023.14.6.1036

**Published:** 2023-10-13

**Authors:** Ulysses Oli, Rose Hosea, B Priya LT Balasubramaniam, Freda Timbi, Gary Nou

**Affiliations:** a Port Moresby General Hospital, Port Moresby, Papua New Guinea.; b National Capital District Provincial Health Authority, Port Moresby, Papua New Guinea.; c World Health Organization Representative Office for Papua New Guinea, Port Moresby, Papua New Guinea.; d National Department of Health, Port Moresby, Papua New Guinea.

## Abstract

**Problem:**

Papua New Guinea (PNG) is situated in the Pacific Ocean and has experienced multiple natural disasters and disease outbreaks. However, PNG lacks the ability to rapidly and systematically deploy trained personnel to provide surge capacity in response to major national disasters. It was therefore decided to establish a national emergency medical team (EMT) in PNG.

**Context:**

PNG’s responses to the 2018 earthquake in the Highlands Region and the coronavirus disease (COVID-19) pandemic required assistance from international EMTs. PNG began developing its own EMT in 2019, coinciding with the development of other Pacific EMTs.

**Action:**

PNG’s EMT project was initiated in 2019 with the creation of a technical working group. By 2021, a focal point had been identified, standard operating procedures had been drafted and training of EMT members had been completed. Pilot deployments of the national EMT members contributed to the COVID-19 response during 2021.

**Outcome:**

Four major challenges were identified during the early phase of PNG’s national EMT development: introducing the concept of EMTs in an EMT-naïve landscape; integrating the national EMT into existing PNG National Department of Health organizational structures; assembling adequate members at short notice to respond to disasters; and securing funding for deployment.

**Discussion:**

Solutions identified for these challenges included strengthening stakeholder involvement through engagement in the development process and participation in technical working groups and consultative group discussions, offering exposure to other Pacific-based EMTs, and creating incentive schemes for EMT members and their place of employment.

Papua New Guinea (PNG) is a lower-middle income country situated in the Pacific Ocean with an estimated population of over 7 million people (according to the 2011 census). (*1*) Like many low- and middle-income countries (LMICs), PNG confronts challenges related to a high disease burden, low socioeconomic status and an inadequately sized health-care workforce. With only 0.7 doctors per 10 000 people, PNG’s physician density is well below the average for LMICs of 8 per 10 000 people. (*2*, *3*)

Papua New Guinea has witnessed multiple disasters and infectious disease outbreaks. These include a major landslide in Simbu (1991), the Rabaul volcano eruption (1994), the Aitape tsunami (1998), a 7.8 magnitude earthquake in the New Guinea Islands (2000), cholera outbreaks (2009), measles outbreaks (2014), a poliomyelitis outbreak (2018) and the PNG Highlands earthquake (2018). (*4*) The health response to national disasters and epidemics has historically been organized by disaster management teams led by the PNG National Disaster Centre and comprised mainly public health personnel from the National Department of Health (NDOH), both at national and provincial levels. When PNG’s front-line health service capacity was exceeded, international emergency medical teams (EMTs) were requested. Assistance from international EMTs was most recently requested during the coronavirus disease (COVID-19) pandemic and served to highlight the deficiencies in PNG’s national emergency medical response provision.

Despite the frequency of these disasters and repeated requests for assistance from international EMTs, a national EMT has never been established in PNG.

## CONTEXT

After the earthquake in Haiti in 2010, the World Health Organization (WHO) launched its global EMT initiative to improve the timeliness and the standard of care offered to affected populations in the aftermath of a disaster by both national and international EMTs. This initiative was also designed to enhance national capacity to coordinate and respond to disasters. (*5*)

Since 2017, regional WHO teams have been assisting Pacific island countries and areas (PICs) to establish their own national EMTs and to navigate the verification process. In 2019, the Fiji Emergency Medical Assistance Team (FEMAT) was verified by WHO. (*6*) EMTs have also been established in the Cook Islands (KukiMAT), Kiribati (KIRIMAT), the Marshall Islands (MIMAT), the Federated States of Micronesia (FSM EMT), the Commonwealth of the Northern Mariana Islands (CNMI EMT), Palau (KLEMAT), Samoa (SEMAT), Solomon Islands (SOLMAT), Tonga (TEMAT), Tuvalu (Tuvalu EMT) and Vanuatu (VANMAT). (*7*)

Several newly established Pacific EMTs have responded to incidents of national and international significance. For example, FEMAT was deployed for a measles outbreak, tropical cyclones and COVID-19 in 2019, and VANMAT provided the health response during Tropical Cyclone Harold in 2020. (*6*) EMTs in the Cook Islands and Tonga aided in COVID-19 preparedness and response efforts. In January 2022, Tonga’s EMT responded to the Hunga Tonga–Hunga Ha’apai volcanic eruption and tsunami and provided care for 381 patients. (*8*)

International EMTs were deployed to PNG in 2018 following the earthquake that struck the Highlands region affecting 544 000 people, of whom 270 000 needed immediate humanitarian assistance. At least 18 000 people were displaced from their homes and the health system quickly became overwhelmed. (*9*) The Australian Medical Assistance Team (AUSMAT) was one of the first international EMTs deployed to the region to assist with the response and to support national efforts to deploy several EMTs. These teams comprised emergency physicians, paramedics, acute care staff and public health officers, as well as WHO/UNICEF officers who were quickly assembled by the medical controller of the region. The assistance of several international EMTs was also requested during the COVID-19 pandemic when the health system in PNG was again overwhelmed.

## ACTION

In August 2019, WHO had initial discussions with NDOH regarding the establishment of a national EMT for PNG. Following these discussions, and with additional support from the Australian Department of Foreign Affairs and Trade (DFAT), the Australian Department of Health and the Australian National Critical Care and Trauma Response Centre (NCCTRC), a technical working group was created. This led to the development of an operational concept for the PNG EMT that set out the essential components of the team and its operations, such as the team’s structure; staffing, equipment and training needs; and deployment procedures. The overarching objective was to gradually transition away from the reliance on international EMTs, and instead establish a fully functional national EMT coordination cell capable of rapidly deploying a medical team.

**Fig. 1:**
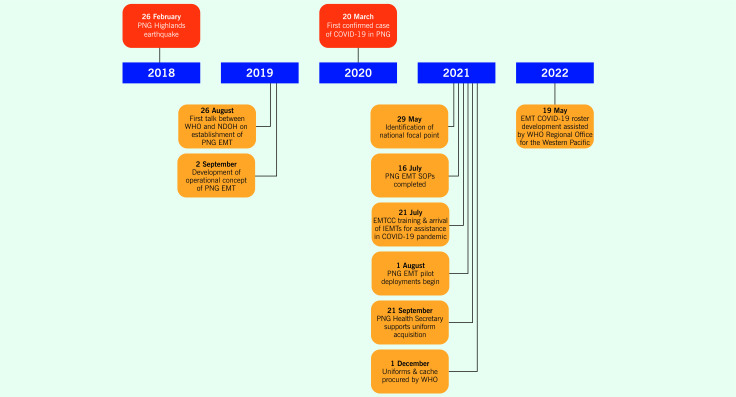
Timeline of key events in the establishment of an emergency medical team in Papua New Guinea

In 2021, a PNG EMT focal point was identified. Focal point staff attended training at the NCCTRC in Darwin to learn how to coordinate the response to a surge in health-care needs resulting from increased morbidity or damage to existing capacities. (*10*) In addition, COVID-19 standard operating procedures (SOPs) for the PNG EMT were finalized, and initial pilot deployments were carried out in response to COVID-19 surges in several provinces. (*11*) A cache of EMT equipment and consumables and uniforms was also procured (**Fig. 1**). (*12*)

[**insert** Figure 1]

The recruitment of EMT members followed a stepwise process, beginning with an initial call-out to the emergency medicine and anaesthesia fraternities, the allied health-care community, and biomedical as well as infection prevention and control (IPC) officers. Those who expressed an interest in volunteering to be part of the team were subsequently invited to participate in a screening and selection process managed by the PNG EMT lead, which resulted in 34 members being selected.

In October 2021, following consultations with senior members of AUSMAT, PNG EMT members were given further training in essential skills, such as basic life support and advanced cardiovascular life support. Team members also completed Major Incident Medical Management and Support training, an Australian-based course on handling a major health disaster.

During 2021 and 2022, PNG’s EMT had 11 deployments. Four were to provinces within PNG that requested assistance with their clinical services during a surge of COVID-19 cases. The remaining seven deployments were dedicated to peacetime activities and involved delivering week-long, just-in-time COVID-19 training to provincial and district hospital staff. Training materials were developed in consultation with the WHO case management team, NDOH clinicians and visiting clinical experts and covered topics such as the appropriate use of oxygen, maintenance of oxygen delivery equipment and oxygen concentrators; COVID-19 health-care pathways and clinical management; and IPC measures. Selected EMT members (senior clinicians, biomedical focal points and IPC officers) were trained to deliver these trainings. The deployments typically involved teams of five to six individuals, comprising a medical doctor, a nurse, a biomedical officer, an IPC officer and a WHO case management consultant. The training sessions also provided an opportunity to raise EMT awareness among senior provincial managers and staff of provincial health authorities (PHAs).

## OUTCOME

The four main challenges surrounding the establishment and deployment of the PNG EMT can be summarized as follows:

unfamiliarity with the EMT concept leading to lack of cooperation among health stakeholders;
integration of the EMT into existing organizational structures;
availability of team members at short notice for deployment; and
availability of funding for deployment.

### Introducing the concept of emergency medical teams

Introducing the EMT concept in an EMT-naïve country proved more challenging than anticipated. The difficulties in getting the full cooperation of PHAs, hospital sectional heads and clinicians during both disaster and peacetime deployments illustrated that many did not fully understand the role of an EMT. Volunteers were mainly recruited from Port Moresby, the nation’s capital, whereas the yield of volunteers from other parts of the country was poor. Furthermore, many clinicians were not given necessary approvals by their sectional heads to be part of the EMT.

### Integrating the emergency medical team into existing organizational structures

To be effective and sustainable, the national EMT’s framework needs to be integrated into existing national health structures. The PNG EMT was developed during the COVID-19 pandemic under the National Control Centre as part of the COVID-19 response. However, the subsequent integration of the PNG EMT into the NDOH structure has been slow due to uncertainty around which of NDOH’s three major subdivisions – the Corporate Services Division, the Public Health Division and the Medical Standards Division – would be the most appropriate host. Given the overlap between their services and those provided by an EMT (which include both clinical and public health components), both the Public Health and the Medical Standards Divisions were proposed as potential hosts. This decision had important implications for the integration process, as it governed how PNG’s EMT will be activated during a crisis, what will be the trigger points for activation, and how it will be funded. Other external factors, such as the restructuring of NDOH in the wake of the 2021 launch of the National Health Plan 2021–2030, have further impacted the pace of the integration process.

### Assembling members at short notice for disaster response

For an EMT to serve its purpose, there needs to be a prompt, organized and coordinated deployment of trained professionals in response to an incident or emergency. This was not observed during the initial deployments of the PNG EMT to provinces that were experiencing COVID-19 case surges. Although there were urgent calls for help from affected provinces, it proved difficult to assemble sufficient numbers of EMT personnel to respond to requests for assistance. Sectional heads were reluctant to allow their staff to be released for EMT deployments, and the urgency of the deployment was not recognized by various PHAs or the EMT personnel themselves. This resulted in frequent postponements and even cancellations of scheduled deployments. In some cases, deployment delays of up to 1 week were reported, which clearly fell short of WHO’s recommended time frame of deployment within 24 hours for a Type 1 mobile EMT response in the event of disasters or emergencies. (*5*)

### Funding for deployment

The initial deployments of the PNG EMT were funded by nongovernmental agencies. During the COVID-19 Delta surge, PNG EMT deployments were fully partner-funded by WHO and other partners such as Australia’s DFAT, which – working through the PNG-Australia Transition to Health (PATH) programme – oversaw all operational and logistical costs. Domestic funding mechanisms have not yet been put in place, which means that PNG is currently not able to fund its own EMT programme. Continued reliance on funding from nongovernmental agencies risks causing delays in deployment as formal requests for funding must go through partner-specific protocols.

## Discussion

The process of establishing an EMT in PNG has not been without its challenges, but some valuable lessons have been learned. Strengthening stakeholder involvement in EMT development and operations was identified as key to addressing some of the initial barriers to the introduction of the concept of a national EMT. Based on our experience, we would recommend engaging with senior executive members and clinical and sectional heads of the NDOH and PHAs, with a view to raising awareness of the EMT concept and how it will function during a disaster. As part of these awareness-raising activities, it is important to highlight how EMTs can benefit the national disaster response as well as PHAs during a disaster. Awareness activities also present an opportunity to forge partnerships with provincial hospitals to ensure effective communication and coordination during emergencies, as well as to facilitate mobilization of EMT personnel. It can also be helpful to invite other established EMTs, particularly from the Pacific region, to present their experiences in establishing a national EMT. This can provide insights into the usefulness of a national EMT and into the challenges experienced by other teams in similar settings.

In addition to engaging with stakeholders through various EMT concept awareness activities, we would suggest inviting representatives from each of the above-mentioned key stakeholder groups to join technical working groups and/or to participate in consultative group discussions. This facilitates a two-way conversation and provides feedback and learnings from previous emergency responses that could be used to improve future responses. This form of stakeholder engagement may also pave the way for meaningful discussions about the positioning of the EMT within the health system, as well as the protocols for activation.

Engagement with other partners and agencies involved in the emergency response system – which in PNG includes the National Disaster Centre managed by the defence forces, St John’s Ambulance and various other health cluster partners – can also help overcome some of the challenges associated with integration. The roles and responsibilities of each group in the EMT would need to be determined, agreed upon and communicated. It is recommended that EMT leads work to secure longer-term partnerships and working relationships with local and international organizations to ensure ongoing support for its cause, particularly in terms of funding and technical assistance.

Identified solutions to the difficulties in assembling an adequately sized EMT at short notice include creating awareness of the benefits of the EMT concept among senior management and sectional heads both at the NDOH and PHA levels. This will ensure that staff who are part of the EMT will be released when required by their supervisors within the time frames required. Recommended strategies for improving recruitment include providing incentives for health-care professionals to join the PNG EMT, such as career development opportunities, professional recognition and financial compensation. NDOH can also develop and implement policies that mandate participation in the PNG EMT as part of the job requirements for health-care workers. At the PHA level, the PNG EMT can provide training and support to hospital staff to ensure they are aware of the importance of the role of the PNG EMT and encourage their participation. Rostering of EMT-specific training for hospital staff could be introduced to equip EMT members with the necessary skills to support the PNG EMT response during deployments. In addition, incentives can be offered to hospital staff who participate in PNG EMT deployments, such as additional compensation or time off in lieu. Branding the national EMT as a prestigious group, one that staff may be proud to be part of, can also improve its attractiveness and could increase recruitment figures.

Public awareness campaigns to educate the public about the importance of an EMT and its role in responding to emergencies may also be helpful. Several published studies have found that lack of public awareness was a factor that directly hindered implementation of an emergency medical services system. (*13*, *14*) Awareness is key to obtaining stakeholder buy-in to the initiative. Partnerships with other nongovernmental organizations can also be used as an avenue for increasing awareness and support for the EMT initiative.

In conclusion, although the establishment of the PNG EMT has been challenging, it is expected to become a sustainable entity within PNG’s NDOH. As a result, PNG will join a growing number of countries in the Pacific region that have successfully established their own EMTs. It is hoped that the challenges and solutions outlined in this article can assist other LMICs that are considering setting up a national EMT.
